# Dual antiplatelet pre-treatment with aspirin and ticagrelor in ACS patients undergoing unplanned aortocoronary bypass surgery

**DOI:** 10.1007/s00392-025-02629-0

**Published:** 2025-03-12

**Authors:** Christian Salbach, Mustafa Yildirim, Rebecca Gulba, Barbara Ruth Milles, Moritz Biener, Matthias Mueller-Hennessen, Hauke Hund, Norbert Frey, Evangelos Giannitsis

**Affiliations:** Department of Internal Medicine III, Cardiology, University Hospital of Heidelberg, Im Neuenheimer Feld 410, 69120 Heidelberg, Germany

**Keywords:** Acute coronary syndrome, Coronary artery bypass grafting, Dual antiplatelet therapy, Real-world evidence

## Abstract

**Background:**

Major bleedings following coronary artery bypass grafting (CABG) have significant implications on outcomes in acute coronary syndrome (ACS) patients. Owing fears of fatal bleedings in case of urgent CABG, current guidelines recommend a cessation of P2Y_12_ receptor antagonists (P2Y_12_-RA) before cardiac surgery and opt against routine pre-treatment with a P2Y_12_-RA before coronary angiography (CA). However, sparse information exists outside randomized trials on the frequency of urgent CABG and the consequences of inappropriately long cessation of P2Y_12_-RA treatment in patients presenting with ACS.

**Methods:**

In this observational single-center study, ACS patients presenting to an emergency department requiring a CABG were recruited consecutively during a 2-year enrolment period. Baseline characteristics, CABG-related bleedings and all-cause mortality were collected from electronical medical records and related to the timing of CABG and P2Y_12_-RA cessation.

**Results:**

A total of 1,502 ACS patients were included, herein 102 (6.8%) underwent urgent CABG. The majority (76.5%) received a routine P2Y_12_-RA pre-treatment predominantly ticagrelor in addition to low-dose aspirin before CA. 31 (30.4%) developed a CABG-related bleeding event. Bleeding probability was highest (HR: 4.77, 95%CI 2.20–10.37, *p* = 0.0001) when CABG was performed within 24 h after administration of dual anti-platelet therapy (DAPT). Despite high utilization rates of DAPT pre-treatment and high prevalence of CABG-related major bleedings, no fatal bleedings occurred.

**Conclusions:**

Need of urgent CABG in ACS is infrequent and does not result in an excess of mortality. However, cessation of ticagrelor for at least 48 h before CABG is recommended to minimize rates of CABG-related bleedings.

**Graphical Abstract:**

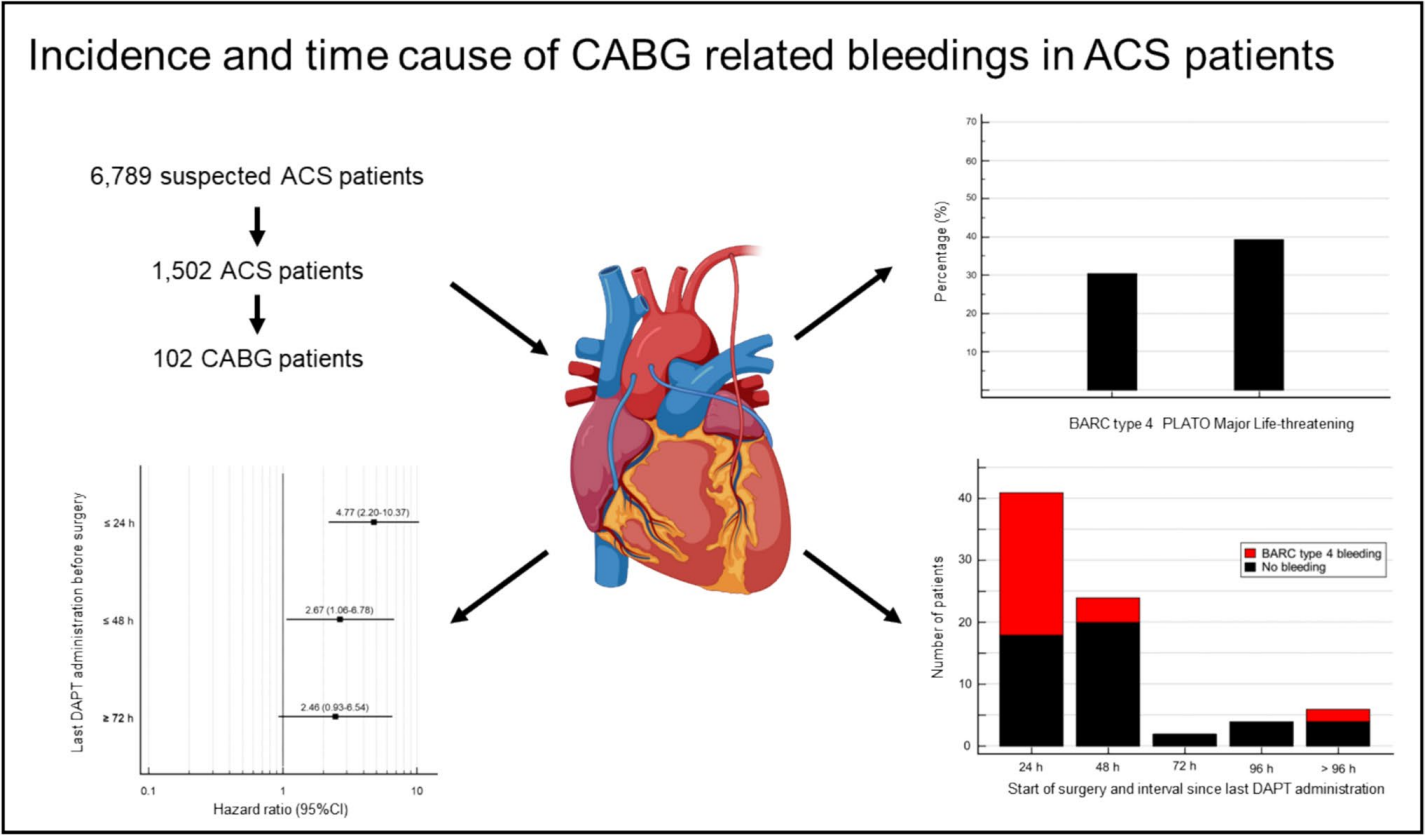

**Supplementary Information:**

The online version contains supplementary material available at 10.1007/s00392-025-02629-0.

## Introduction

Administration of dual antiplatelet therapy (DAPT), consisting of aspirin and a potent P2Y_12_ receptor antagonist (RA) has been shown to reduce thrombotic risk for patients presenting with an acute coronary syndrome (ACS) [1, 20]. However, the timing for initiating DAPT is under debate. The 2020 European Society of Cardiology (ESC) Guidelines on non-ST-segment elevation acute coronary syndrome (NSTE-ACS) and the ESC 2023 Guidelines on ACS advocate against a P2Y_12_-RA pre-treatment in patients with NSTE-ACS planned for an early invasive strategy until coronary anatomy is known [1, 2]. This recommendation is backed by findings from the Intracoronary Stenting and Antithrombotic Regimen Rapid Early Action for Coronary Treatment (ISAR REACT) 5 trial [12] and the A Comparison of Prasugrel at the Time of Percutaneous Coronary Intervention or as Pre-treatment at the Time of Diagnosis in Patients with Non-ST-Elevation Myocardial Infarction (ACCOAST) trial [9] and driven by fear of a major or fatal bleeding event in case patients require an emergency aortocoronary bypass grafting (CABG) and cannot pause the P2Y_12_-RA adequately long to partially restore platelet function. Sparse data are available on the frequency and sequelae of emergent CABG surgery in patients with ACS. In a sub investigation of the Platelet Inhibition and Patient Outcomes (PLATO) trial for ACS patients who received a CABG, ticagrelor or clopidogrel treatment was withheld for 24–72 h in case of ticagrelor and 5 days in case of clopidogrel and the drugs were re-started as soon as possible after surgery and before discharge. The majority of major bleedings occurred in patients where P2Y_12_-RAs could not be paused for 3 days or longer but without a significant difference regarding the risk of CABG related bleedings or an excess of fatal or life-threatening bleedings between clopidogrel and ticagrelor. On the other hand, ticagrelor treatment was associated with a more than 50% reduction in total cardiovascular mortality during 12-months of follow-up, mainly due to reduction in sepsis-related death. [6, 18] At present, 2017 ESC Guidelines on Myocardial Revascularization recommend a cessation of surgery procedures including non-emergent cardiac surgery patients for at least 3 days in case of ticagrelor, 5 days in case of clopidogrel and 7 days in case of prasugrel with a class IIa, B recommendation. [10] In addition, 2021 guidelines of the American Heart Association and the American College of Cardiology (AHA/ACC) on Myocardial Revascularization recommend the continuation of aspirin medication until the time of surgery but the discontinuation of clopidogrel or ticagrelor for at least 24 h in patients referred for urgent CABG. [7] In real-world, sparse information exists outside randomized trials on the frequency of emergent CABG and the consequences of inappropriately long cessation of P2Y_12_-RA treatment in patients presenting with ACS. This study aimed to assess the real-world frequency of major bleedings in ACS patients who received routine DAPT pre-treatment and underwent CABG within 30 days, with a particular focus on emergency CABG within the initial days after admission.

## Methods

### Data availability

The data underlying this article will be shared on reasonable request to the corresponding author.

### Study design

**Fig. 1:**
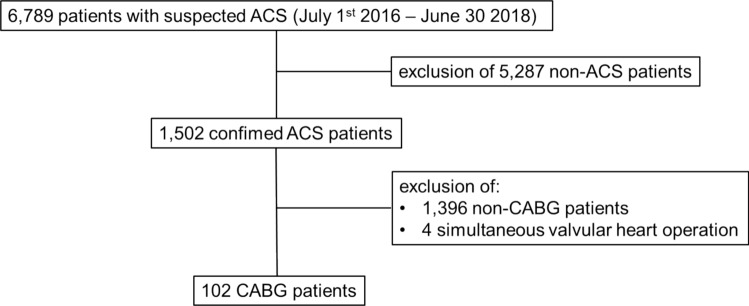
Consort diagram for included patients. ACS, acute coronary artery syndrome; CABG, coronary artery bypass graft

This retrospective observational single-center study consecutively enrolled patients with suspected ACS presenting to the chest pain unit (CPU) of the University Hospital of Heidelberg during a 2-year recruitment period between 1st of July 2016 and 30th of June 2018. The study cohort and local CPU organization was already described earlier [11, 19]. For this analysis, only patients with CABG within 30 days after admission to CPU were included. Patients with planned CABG, or combined valvular heart surgery were not considered (Fig. 1). Acute myocardial infarction (MI) was diagnosed by treating physicians based on the diagnostic criteria of the 3rd or 4th universal MI definition [16, 17]. All CPU diagnoses were retrospectively re-adjudicated for research purposes by two cardiologists, and a third cardiologist in case of discordance. At the time of study recruitment, DAPT pre-treatment with 180 mg ticagrelor loading followed by 90 mg BID in combination with 250 mg aspirin loading followed by 100 mg OD represented the default strategy across the entire spectrum of ACS. Clopidogrel was preferred for patients with an initially normal highly sensitive cardiac troponin (hs-cTn) levels while prasugrel was administered only as an alternative to ticagrelor in case of label restrictions or warnings against ticagrelor. Several findings from this registry have been published earlier [14, 20]. Outcomes for 90-day all-cause mortality were collected from medical records, phone calls, questionnaires and from death certificates. The study was approved by the local ethical committee of University of Heidelberg and was conducted in accordance with the 1964 Declaration of Helsinki and its later amendments. Informed consent was waived by local ethical committee.

### Definitions

CABG-related bleedings were defined per Bleeding Academic Research Consortium (BARC) type 4 bleeding or PLATO major life-threatening bleeding definitions [8]. A BARC type 4 bleeding was defined as (a) a perioperative intracranial bleeding within 48 h, (c) re-operation after closure of the sternotomy incision for the purpose of controlling bleeding (d) transfusion of ≥ 5 units packed red blood cell (PRBCs) or whole blood within 48 h period. [8] A PLATO major life-threatening bleeding was defined as one of the following criteria: Fatal bleeding resulting in death, intracranial bleeding, intrapericardial bleeding with cardiac tamponade, bleeding resulting in hypovolemic shock or severe hypotension requiring vasopressors or surgery, clinical overt or apparent bleeding associated with a decrease in hemoglobin > 5 g/dl, requiring a transfusion of ≥ 4 units of whole blood or PRBCs. [8] The time, given in hours, from last intake of the P2Y_12_-RA was retrieved from the anesthesia protocols and electronic medical records.

For comparison of ischemic and bleeding risks, the original Global Registry of Acute Coronary Events (GRACE) 1.0 score and the Predicting Risk of Bleeding Complications in Patients Undergoing Dual Antiplatelet Therapy (PRECISE-DAPT) score were calculated retrospectively [3, 4]. If a previous bleeding event had not been registered, this item was assigned zero points for calculation of PRECISE-DAPT score.

### Statistical and data analyses

Continuous variables were tested for normal distribution and were presented either as means with 95% confidence intervals (CI), or as medians with 25th/75th percentiles (interquartile range, IQR). The normality of data distribution was assessed by the Kolmogorov–Smirnov test. Groups were compared using the χ2 test for categorical variables and the Mann–Whitney U test for continuous variables. Kaplan–Meier analyses were performed and groups were compared using the Log-Rank test. A multivariate Cox proportional hazards regression was performed to determine predictors for bleeding events. The proportional hazards assumption was tested using the Grambsch and Therneau method. All hypothesis testing was two-tailed and p-values less than 0.05 were considered statistically significant. All statistical analyses were performed using MedCalc 20.111 (MedCalc Software bvba, Ostend, Belgium). The first and corresponding author had full access to all the data in the study and take responsibility for its integrity and the data analysis.

## Results

Among 6789 patients presented to the CPU between 1st June 2016 and 30th June 2018, a total of 1,502 was diagnosed with an ACS. Of these, 1396 patients were excluded from the present analysis as they did not require cardiac surgery within 30 days, 4 patients were excluded because of simultaneous valvular heart surgery, resulting in a total of 102 patients (median age 70 years (IQR 61–76), 88.2% male) receiving CABG within 30 days after admission to the CPU. Among these 102 patients, nine (8.8%) had a diagnosis of ST-segment elevation myocardial infarction (STEMI). Seventy cases (68.6%) had a Non-ST-elevation myocardial infarction (NSTEMI) and twenty-three patients (22.5%) had an unstable angina (UA). The most frequent cardiovascular risk factors were arterial hypertension (75.3%), hypercholesterolemia (59.8%) and diabetes mellitus (41.0%). Mean GRACE 1.0 score was 118 points (SD 33.2) and mean PRECISE-DAPT score was 19 points (SD 9.9). A total of 78 (76.5%) were on DAPT therapy before CABG. Baseline characteristics for patients who underwent CABG surgery are shown in supplement table S1. Median time from admission to CPU until CABG surgery was 3 days (IQR 1–9). Depending on the ACS subtype, time from CPU admission to CABG procedure varied. Time to CABG was 1 day (IQR 0–11) in case of STEMI, 2 days (IQR 1–6) for NSTEMI and 8 days (IQR 4–12) for UA. Rates of urgent CABG procedures within 24 and 48 h after admission were 10.8% and 15.7%.

### Antiplatelet and antithrombotic regimes

At the CPU of the University Hospital of Heidelberg, pre-treatment before invasive procedure is the default strategy in clinically confirmed ACS. Prasugrel is not preferred due to label restrictions and warnings that reduce the number of eligible patients, whereas ticagrelor can be used in a substantially higher proportion of patients with less prevalent label restrictions [20]. As such, prasugrel was only administered in one patient (0.9%). Not unexpectedly, low-dose aspirin and ticagrelor was the most prevalent DAPT regimen (63.2%), followed by aspirin and clopidogrel (12.7%). Other antiplatelet regimens were infrequent and included aspirin monotherapy (10.8%), dual (DAT), or triple therapy (TAT) 4.9% each, or oral anticoagulation monotherapy 2.9%.

### Outcomes and bleeding events

**Fig. 2:**
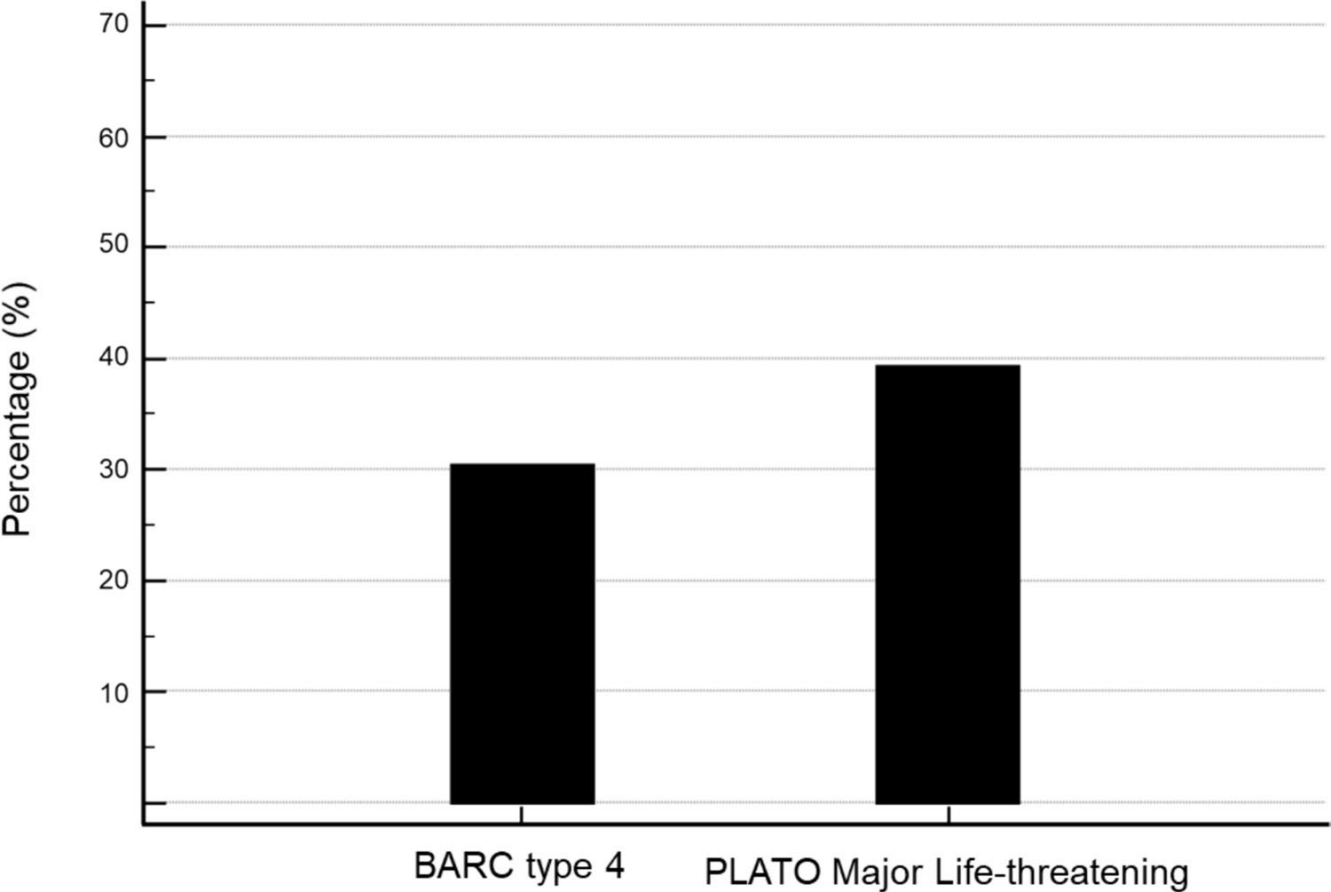
Prevalence of bleeding events in CABG patients according to BARC type 4 and PLATO Major Life-threatening classification. CABG, coronary artery bypass graft; BARC, Bleeding Academic Research Consortium; PLATO, PLATelet inhibition and patient Outcomes

Bleeding events were classified according to the BARC CABG-related bleeding (BARC type 4) and the PLATO major life-threatening bleeding classifications [8]. Among 102 CABG patients, a total of 31 (30.4%) developed a BARC type 4 bleeding and 40 (39.2%) a PLATO major life-threatening bleeding event (Fig. 2). Baseline characteristics for CABG patients classified by the presence or absence of a BARC type 4 bleeding are shown in Table 1. Baseline characteristics differed in regard to preoperative hemoglobin, hematocrit, left ventricular ejection fraction, GRACE- and PRECISE DAPT-score. Among patients with a BARC type 4 bleeding event median application of red blood cell concentrates (RBC) was 6 units (IQR 5–8) and median application of platelets was 2 units (IQR 2–4). The median duration of the CABG procedure was longer in patients who developed a BARC type 4 bleeding event 245 min (IQR 211–281) vs. 215 min (IQR 181–257), *p* = 0.0277. Within 90 days, all-cause mortality was higher among BARC type 4 bleeding patients 4 (12.9%) vs. 2 (2.8%), *p* = 0.0475. Reasons for all-cause mortality was a sepsis in all 4 cases, occurring between day 14 and 44 post surgery. None of the patients died as a direct result to a fatal bleeding event.

**Table 1:** Baseline characteristics for CABG patients with a BARC type 4 bleeding event compared to patients without BARC type 4 bleeding

Variables	BARC-type 4 *n* = 31	No-bleeding event *n* = 71	p-value
Age, y, median (IQR)	73 (66–76)	69 (59–74)	0.0824
Sex (female), *n* (_%all_)	7 (22.6)	5 (7.0)	0.0258
BMI, kg/m^2^, median (IQR)	26.3 (24.7–29.9)	28.1 (2–30.3)	0.0928
Diabetes mellitus, *n* (_%all_)	11 (35.5)	30 (42.3)	0.5233
Arterial hypertension, *n* (_%all_)	24 (77.4)	49 (69.0)	0.3891
Dyslipidemia, *n* (_%all_)	18 (58.1)	34 (47.9)	0.3467
Active smoker, *n* (_%all_)	4 (12.9)	15 (21.1)	0.3289
Family history of CAD, *n* (_%all_)	8 (25.8)	22 (31.0)	0.5993
History of CAD, *n* (_%all_)	11 (35.5)	19 (26.8)	0.3762
Atrial fibrillation, *n* (_%all_)	2 (6.5)	8 (11.3)	0.4541
Former stroke or TIA, *n* (_%all_)	2 (6.5)	4 (5.6)	0.8724
Obstructive PD, *n* (_%all_)	0 (0)	7 (9.9)	0.0715
Serum creatinine, mg/dl, median (IQR)	1.02 (0.87–1.41)	0.94 (0.83–1.07)	0.0896
Leukocytes, /nL, median (IQR)	7.6 (6.5–10.3)	8.2 (7.1–9.8)	0.7885
C-reactive protein mg/L, median (IQR)	2.9 (1.9–10.4)	2.1 (1.9–8.0)	0.3912
Hemoglobin g/dl, median (IQR)	13.1 (12.2–14.3)	14.7 (13.6–15.4)	< 0.0001
Hematocrit l/l, median (IQR)	0.37 (0.35–0.42)	0.43 (0.40–0.45)	0.0001
Platelets /nL, median (IQR)	234 (203–284)	224 (194–274)	0.2684
INR, median (IQR)	1.07 (1.02–1.14)	1.04 (0.98–1.10)	0.1819
Hs-cTnT, ng/L, median (IQR)	71 (26–213)	33 (17–112)	0.0873
LVEF, %, median (IQR)	40 (30–55)	53 (40–55)	0.0251
GRACE 1.0 score, median (IQR)	128 (112–162)	109 (96–135)	0.0050
PRECISE DAPT score, median (IQR)	24 (15–34)	18 (11–24)	0.0098
Operation procedure			
Number of CABG grafts			0.3356
One-vessel, *n* (_%all_)	2 (6.5)	4 (5.6)	
Two-vessel, *n* (_%all_)	11 (35.5)	21 (29.6)	
Three-vessel, *n* (_%all_)	12 (38.7)	25 (35.2)	
Four-vessel, *n* (_%all_)	5 (16.1)	17 (23.9)	
Five-vessel, *n* (_%all_)	1 (3.2)	4 (5.6)	
CABG graft selection			0.7570
Arterial and venous, *n* (_%all_)	26 (83.9)	57 (80.3)	
All-arterial, *n* (_%all_)	4 (12.9)	12 (16.9)	
All-venous, *n* (_%all_)	1 (3.2)	2 (2.8)	
Erythrocyte concentrates, n, median (IQR)	6 (5–8)	2 (0–3)	< 0.0001
Platelet concentrates, n, median (IQR)	2 (2–4)	2 (0–2)	< 0.0001
Operation duration, minutes, median (IQR)	245 (211–281)	215 (181–257)	0.0227
Pre-operative antiplatelet therapy			
DAPT, *n* (_%all_)	29 (93.5)	49 (69.0)	0.0075
Last DAPT administration before CABG, h, median (IQR)	13 (6–18)	26 (14–37)	0.0051
Aspirin/Ticagrelor, *n* (_%all_)	28 (90.3)	36 (50.7)	0.0002
Aspirin/Prasugrel, *n* (_%all_)	0 (0)	1 (1.4)	0.5088
Aspirin/Clopidogrel, *n* (_%all_)	1 (3.2)	12 (16.9)	0.0580
Aspirin mono, *n* (_%all_)	0 (0)	11 (15.5)	0.0210
Dual therapy*, *n* (_%all_)	1 (3.2)	4 (5.6)	0.6062
Triple therapy, *n* (_%all_)	1 (3.2)	4 (5.6)	0.6062
OAC mono, *n* (_%all_)	0 (0)	3 (4.2)	0.2477

BARC, Bleeding Academic Research Consortium; BMI, body mass index; CABG, coronary artery bypass graft; CAD, coronary artery disease; DAPT, dual antiplatelet therapy; GRACE, Global Registry of Acute Coronary Events; hs-cTnT, highly sensitive cardiac troponin T; INR, internationalized normalized ratio; IQR, interquartile range; LVEF, left ventricular ejection fraction; MI, myocardial infarction; OAC, oral anticoagulation; PD, pulmonary disease; PRECISE DAPT, PREdicting bleeding Complications In patients undergoing Stent implantation and subsequent Dual Antiplatelet Therapy; TIA, transient ischemic attack; y, years

^*^dual therapy: oral anticoagulation plus clopidogrel

The majority of BARC type 4 bleeding patients received a DAPT therapy before CABG (93.5%, *p* = 0.0075), one patient developed a BARC type 4 bleeding during DAT and one patient while on TAT, each with paused anticoagulant. Within the BARC type 4 bleeding group, the majority of the patients 28 (90.3%) were on DAPT with aspirin/ticagrelor, only one patient was on aspirin/clopidogrel. In patients receiving a DAPT regime prior to CABG, median time of last DAPT administration before surgery was 13 h (IQR 6–18) within BARC type 4 group and 26 h (12–37), *p* = 0.0051 within the non-bleeding group. In patients without a BARC type 4 bleeding event DAPT was prevalent in 49 (69%) of the cases. Most prevalent DAPT regime was aspirin/ticagrelor in 36 (50.7%) followed by aspirin/clopidogrel in 12 (16.9%). In patients without a BARC type 4 bleeding, reasons for all-cause mortality were unknown in one case and an ischemic apoplexy in one case.

**Fig. 3:**
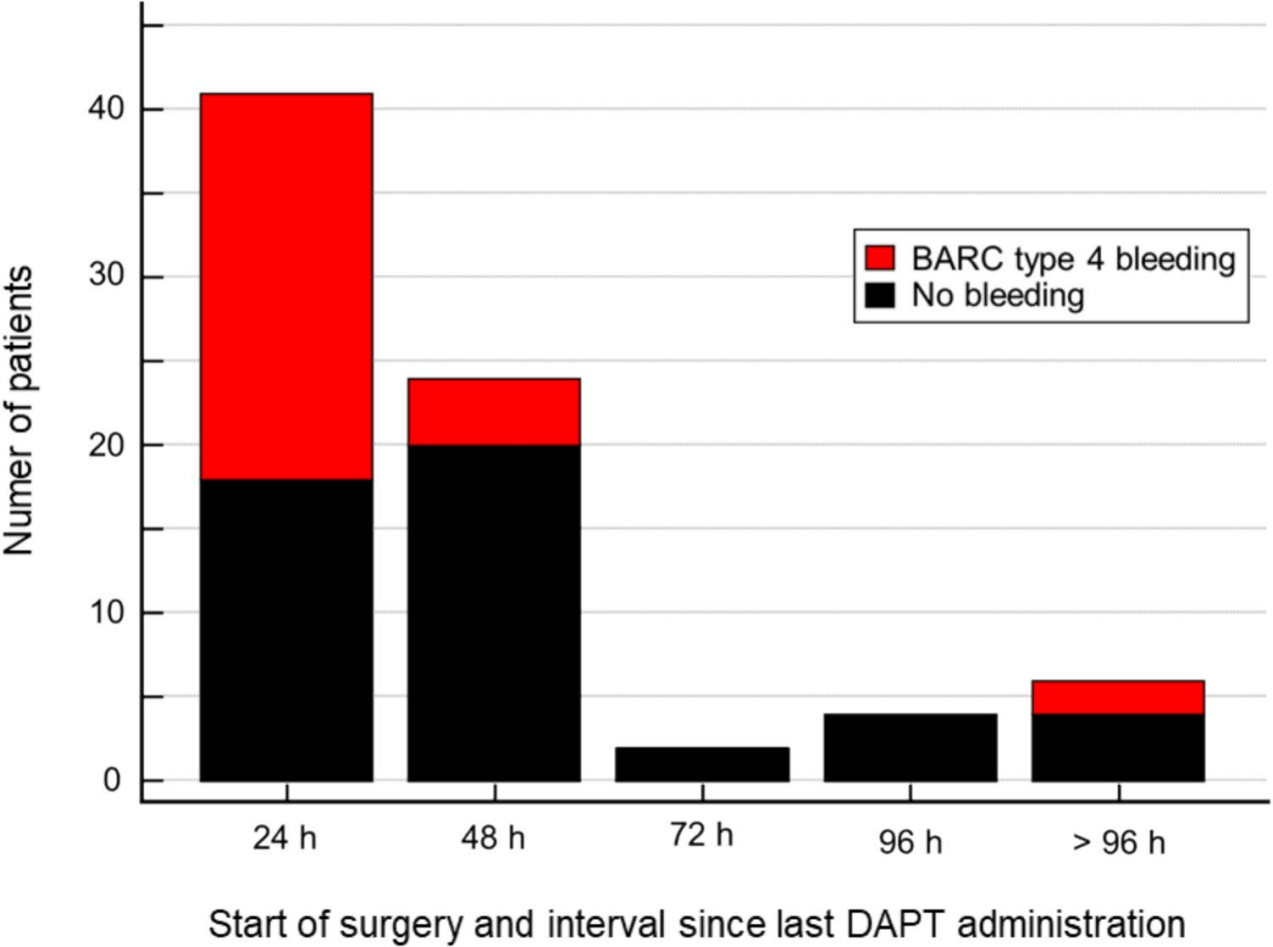
BARC type 4 bleeding events classified by start of surgery and hours since last DAPT administration. BARC; Bleeding Academic Research Consortium

**Fig. 4:**
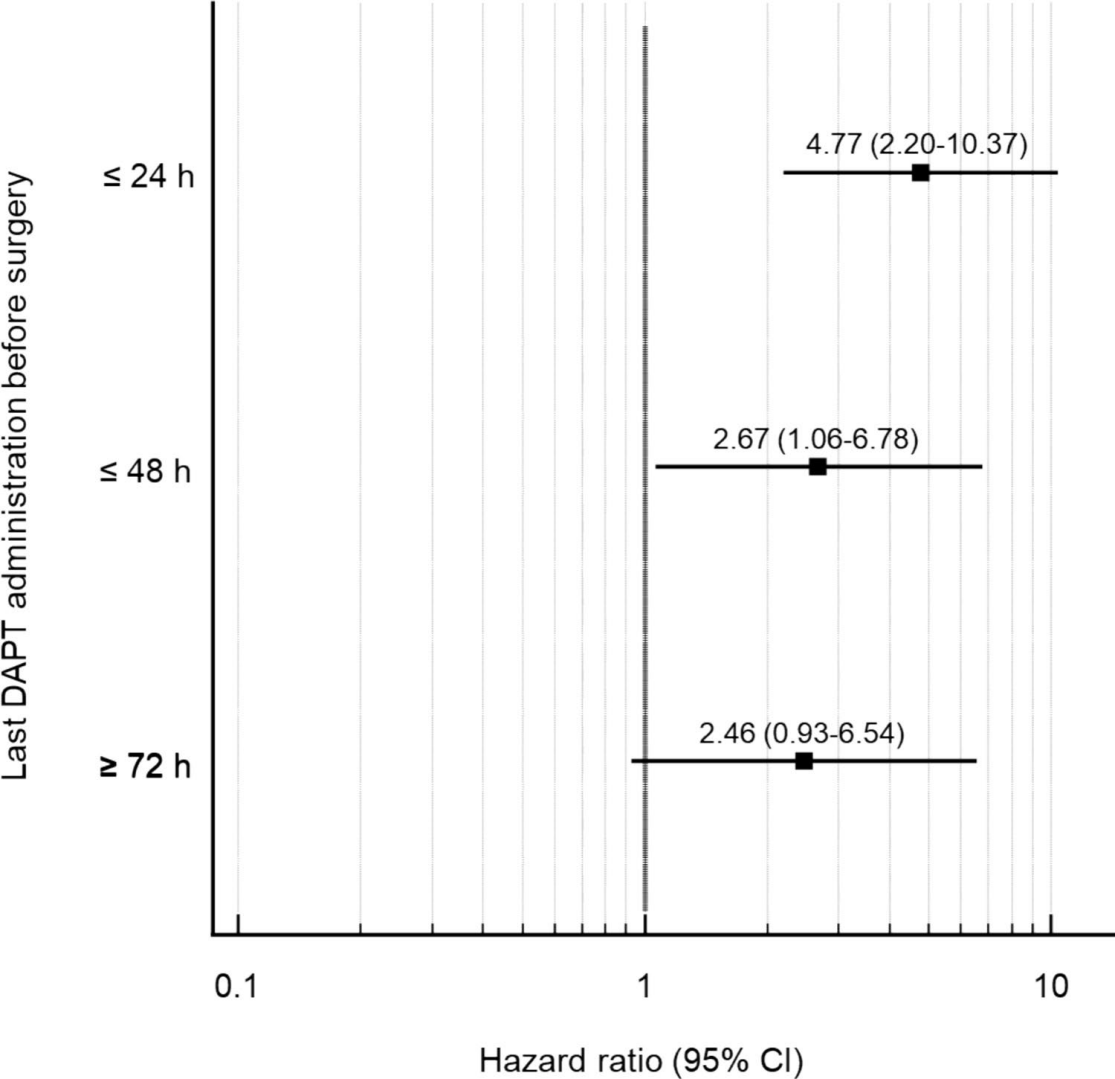
Hazard ratios for CABG-related bleedings (BARC type 4) by last intake of DAPT before surgery compared to patients who did not receive DAPT before surgery. CI, confidence interval; DAPT, dual antiplatelet therapy

79.3% of the bleeding events occurred within patients where DAPT within 24 h before surgery was administered (Fig. 3). The hazard ratio (HR) for a BARC type 4 bleeding event with a DAPT administration within 24 h before surgery was 4.77 (95%CI 2.20–10.37), *p* = 0.0001 Fig. 4. In a Cox-proportional hazard regression model including age, sex, BMI, GRACE and PRECISE DAPT-score as well as pre-operative hemoglobin, the adjusted HR for a BARC type 4 bleeding event in patients with a DAPT administration ≤ 24 h was 5.75 (95%CI 1.94–17.07), *p* = 0.0016 (table S2).

## Discussion

There is an ongoing debate regarding the safety of DAPT pre-treatment in ACS. This debate is largely fostered by fears of life-threatening or fatal bleedings that may be expected when P2Y_12_-RA cannot be paused adequately long in patients who need to undergo emergent cardiac surgery. Guideline recommendation for the management of patients in need of emergent or urgent cardiac and non-cardiac surgery are based on limited evidence, mainly coming from the sub-studies of the pivotal trials on DAPT with ticagrelor (PLATO) and prasugrel (TRITON-TIMI) on patients with CABG. [6, 13] However, a randomized 2 × 2 factorial comparison between ticagrelor and prasugrel with or without pre-treatment is currently not available, as the Dubius trial was stopped prematurely due to futility. [15] Therefore, our findings collected in a consecutive series of patients presenting with suspected ACS are very important to provide real-world evidence on the frequency of emergent CABG and rates of major CABG-related bleedings. Pre-treatment with low-dose aspirin and ticagrelor or less frequently clopidogrel was the default strategy at our institution allowing to provide robust evidence from a 2-year recruitment period in an unselected observational cohort. We applied two established bleeding definitions, i.e. BARC type 4 and PLATO-defined CABG-related bleedings to facilitate comparison with other studies. Due to a meticulous registration of the time from cessation of ticagrelor or clopidogrel to surgery, we were able to provide bleeding complications by precise time intervals. Our study provides four major findings:

Firstly, we found that 6.8% of patients presenting with ACS required CABG-surgery or combined cardiac surgery procedures within 30 days after index presentation for ACS. Among those patients, the median time to CABG was 3 days (ranging from 1 to 9 days). However, rates of urgent CABG within 24 and 48 h after admission were 10.8% and 15.7% indicating that in the majority of patients, CABG surgery may be postponed for a reasonable time to allow recovery of platelet function which is faster after cessation of ticagrelor than clopidogrel [5]. Our findings on overall rates of CABG surgery in ACS as well as on the need of CABG within 24 h agree with findings from the CABG sub-study of the PLATO trial [6]. In the pivotal PLATO trial, patients across the entire spectrum of ACS were randomized to either pre-treatment with low dose aspirin combined with clopidogrel or low-dose aspirin combined with ticagrelor before CA. Consecutively, patients were assigned to an early invasive or conservative treatment. In patients requiring CABG, ticagrelor was paused for at least 3 days if deemed feasible. Overall, 10.2% of patients required CABG surgery, of whom 13.6% of the patients underwent CABG within 24 h after P2Y_12_-RAs were stopped before CABG [6]. Hence, our findings confirm the findings of the randomized PLATO trial and demonstrates a low prevalence of cases where potent P2Y_12_-RA cannot be paused for a period of 3 days or longer.

Secondly, and not unexpectedly, rates of major CABG-related bleedings ranged between 30.4 to 39.2% depending on the bleeding definition applied. Using PLATO major life-threatening bleeding definition 39.2% CABG related bleedings were encountered whereas rates of BARC-4 bleedings were only 30.4%. Our findings are consistent with bleeding rates reported in the PLATO trial (42.6% vs. 43.7%) [6]. Major bleedings occurred more often in patients at higher ischemic risk as indicated by a higher GRACE score (128 points vs 109 points, *p* = 0.0050) and higher bleeding risk as suggested by a higher PRECISE-DAPT score (24 points vs 18 points, *p* = 0.0098). Rates of major bleedings were significantly higher in females, patients with lower hemoglobin values before surgery (13.1 g/dl vs. 14.7 g/dl, *p* < 0.0001), and renal function tended to be worse (creatinine 1.02 mg/dl vs. 0.94 mg/dl, *p* = 0.0896) in patients with bleedings. In addition, median duration of the CABG procedure was longer in patients who developed a BARC type 4 bleedings (245 min (IQR 211–281) vs. 215 min (IQR 181–257), *p* = 0.0277). Finally, the median number of red blood cell concentrates was significantly higher in patients with BARC type 4 bleedings (6 units (IQR 5–8) vs 2 units (IQR 2–4), *p* < 00001). However, only cessation of P2Y_12_-RA, predominantly ticagrelor < 24 h before surgery remained independently predictive for CABG-related bleeding after multivariate adjustment with a 5.7-fold higher risk compared to patients who had not received DAPT treatment before surgery.

Thirdly, we found that rates of BARC-4 bleedings were significantly higher in patients who had received DAPT at any time before cardiac surgery (93.5%). Furthermore, times from the last administration of DAPT to cardiac surgery were significantly shorter (13 vs. 26 h, *p* = 0.0051) among those who experienced CABG-related bleedings. By the time of last DAPT administration, 79.3% and 13.8% of the bleeding events had occurred in patients who had received DAPT within 24 h and 48 h before surgery, with adjusted HR of 5.73 (95%CI 1.89–17.35) and 2.67 (95%CI 1.06–6.79) compared to patients who had not received DAPT before surgery.

Fourthly, overall all-cause mortality rate within 90 days was 5.9% and was higher among BARC type 4 bleeding patients 4 (12.9%) vs. 2 (2.8%), *p* = 0.0475. None of the patients died as a direct consequence of a CABG-related bleeding to a fatal bleeding event. In all four fatal cases, sepsis was identified as the cause of death occurring between day 14 and 44 post surgery.

## Limitations

This study is an observational single-center study over a pre-specified 24-month recruitment period and was not designed to collect information on bleeding events or outcomes in ACS patients undergoing CABG surgery within 30 days from index ACS presentation. Given that this study was a retrospective observational study and not a randomized comparison with an extremely low administration of prasugrel, our results do not allow any conclusions on the safety of pre-loading strategy vs. downstream treatment with DAPT in ACS patients. Our results on rates and timing of CABG were collected from a large-volume PCI center affiliated to an academic setting that provides the entire spectrum of myocardial revascularization procedures including high volumes of emergency cardiac surgeries. Therefore, findings may not be generalized to other geographical regions or hospital settings with restricted access to early PCI or CABG. In addition, the vast majority of patients received DAPT pre-treatment with aspirin and ticagrelor. Therefore, findings may not be generalized to hospitals that refrain from DAPT pre-treatment or combine aspirin with clopidogrel for pre-treatment.

In our cohort, only few patients received clopidogrel pre-treatment and only one case was administered prasugrel for DAPT treatment post PCI but before CABG. Therefore, we refrained from an analysis on this small subgroup due to potential type 2 error. Within our study, patients were enrolled between 2016 and 2018, however, techniques for surgery have evolved over the last years, thus bleeding rates might be even lower with contemporary techniques.

Finally, the majority of the information on CABG procedure and bleeding complications was extracted from electronic files which were screened by health care professionals, i.e. cardiologists in training and licensed study nurses.

## Conclusions

Our findings reflecting clinical practice in a large university hospital suggest that the fear to suffer fatal or life-threatening CABG-related bleedings is not substantiated if patients receive DAPT pre-treatment routinely. Need of urgent CABG surgery within 48 h of index presentation of ACS was infrequent. While CABG-related major bleedings occurred significantly more often in patients with DAPT before surgery, there was no excess risk of death due to bleeding. In particular, no death occurred in patients in whom DAPT could not be paused for at least 72 h to achieve partial recovery of platelet function. Overall, our findings are in agreement with the observations from the randomized PLATO trial and nicely support recommendations of the 2021 AHA/ACC Guidelines on Myocardial Revascularization that promote the discontinuation of clopidogrel or ticagrelor for at least 24 h in patients referred for urgent CABG. [7] The findings of this study also support the recommendation of current ESC guidelines not to administer DAPT in patients with a higher propensity of urgent CABG surgery, which includes STEMI patients and NSTE-ACS patients proceeding to immediate PCI [1].

## Supplementary Information

Below is the link to the electronic supplementary material.

Supplementary file1 (DOCX 16 KB)

## Abbreviations

ACS: Acute coronary syndrome
ACCOAST: A comparison of prasugrel at the time of percutaneous coronary intervention or as pre-treatment at the time of diagnosis in patients with non-ST-elevation myocardial infarction
AHA/ACC: American Heart Association and the American College of Cardiology
BARC: Bleeding Academic Research Consortium (BARC)
CA: Coronary angiography
CABG: Coronary artery bypass grafting
CPU: Chest pain unit
DAPT: Dual antiplatelet therapy
DAT: Dual antiplatelet therapy
DUBIUS: Duration of dual antiplatelet therapy in patients with acute coronary syndrome treated with PCI with second-generation drug-eluting stents: a randomized urgent or balanced decision
ESC: European Society of Cardiology:
GRACE: Global Registry of Acute Coronary Events
Hs-cTn: Highly sensitive cardiac troponin
ISAR-REACT: Intracoronary stenting and antithrombotic regimen: rapid early action for coronary treatment
MI: Myocardial infarction
NSTE-ACS: Non-ST-segment elevation acute coronary syndrome
NSTEMI: Non-ST-segment elevation myocardial infarction
PCI: Percutaneous coronary intervention
PLATO: Platelet inhibition and patient outcomes
PRBCs: Packed red blood cells
RA: Receptor antagonist
RBC: Red blood cell concentrates
STEMI: ST-segment elevation myocardial infarction
TAT: Triple antiplatelet therapy
UA: Unstable angina
