# Supplementary material for: Dual antiplatelet pre-treatment with aspirin and ticagrelor in ACS patients undergoing unplanned aortocoronary bypass surgery
Source: Clin Res Cardiol. 2025 Mar 12;114(6):772–82. doi: 10.1007/s00392-025-02629-0 (PMC12089244; doi:10.1007/s00392-025-02629-0)
Supplement: Supplementary file 1 — Supplementary file1 (DOCX 16 KB) [file 392_2025_2629_MOESM1_ESM.docx]

**Supplementary Information**

**Supplemental tables**

**Supplement Table 1**. Baseline characteristics for CABG patients

| Variables |  |
| --- | --- |
| Age, y, median (IQR) | 70 (61-76) |
| Sex, male, n (_%all_) | 90 (88.2) |
| BMI, kg/m², mean (SD) | 27.8 (3.11) |
| Diabetes mellitus, n (_%all_) | 41 (41.0) |
| Arterial hypertension, n (_%all_) | 73 (75.3) |
| Dyslipidemia, n (_%all_) | 52 (59.8) |
| Active smoker, n (_%all_) | 19 (20.7) |
| Family history of CAD, n (_%all_) | 30 (33.7) |
| Prior MI, n (_%all_) | 19 (18.8) |
| Atrial fibrillation, n (_%all_) | 10 (9.8) |
| Former stroke or TIA, n (_%all_) | 6 (5.9) |
| Obstructive PD, n (_%all_) | 7 (6.9) |
| Serum creatinine, mg/dl, median (IQR) | 0.97 (0.83-1.11) |
| Leukocytes, /nL, median (IQR) | 8.2 (6.7-9.9) |
| C-reactive protein mg/L, median (IQR) | 2.40 (1.00-8.55) |
| Hemoglobin g/dl, median (IQR) | 14.3 (13.2-15.2) |
| Hematocrit l/l, median (IQR) | 0.42 (0.38-0.45) |
| Platelets /nL, median (IQR) | 228 (197-278) |
| Hs-cTnT, ng/L, median (IQR) | 43.5 (18-149) |
| GRACE-1.0 score, mean (SD) | 118 (33.2) |
| PRECISE DAPT-score, mean (SD) | 19 (9.9) |
| LVEF, (%), median (IQR) | 50 (40-55) |
|  |  |
| Pre-operative antiplatelet therapy |  |
| DAPT, n (_%all_) | 78 (76.5) |
| Aspirin/ticagrelor, n (_%all_) | 64 (63.2) |
| Aspirin/prasugrel, n (_%all_) | 1 (1.0) |
| Aspirin/Clopidogrel, n (_%all_) | 13 (12.7) |
| Aspirin mono, n (_%all_) | 11 (10.8) |
| Dual therapy*, n (_%all_) | 5 (4.9) |
| Triple therapy, n (_%all_) | 5 (4.9) |
| OAC mono, n (_%all_) | 3 (2.9) |
|  |  |

*dual therapy: oral anticoagulation plus clopidogrel. Abbreviations: BMI, body mass index; CRP, c-reactive protein; CAD, coronary artery disease; DAPT, dual antiplatelet therapy; GRACE, Global Registry of Acute Coronary Events; hs-cTnT, highly sensitive troponin T; IQR, interquartile range; LVEF, left ventricular ejection fraction; MI, myocardial infarction; OAC, oral anticoagulation; PD, pulmonary disease; PRECISE DAPT, PREdicting bleeding Complications In patients undergoing Stent implantation and subsequent Dual Antiplatelet Therapy; TIA, transient ischemic attack; y, years.

**Table S2.** **Cox proportional hazard regression model for BARC type 4 bleeding events.**

| Covariate | aHR (95%CI) | p-value |
| --- | --- | --- |
| DAPT administration ≤ 24 h before CABG | 5.7259 (1.8900-17.3469) | 0.0020 |
| Age, years | 1.068 (0.9695-1.0875) | 0.3660 |
| Sex, female | 1.3284 (0.4692-3.7607) | 0.5907 |
| BMI, kg/m² | 0.9960 (0.8890-1.1160) | 0.9453 |
| GRACE-score | 1.0077 (0.9933-1.0222) | 0.2970 |
| Hemoglobin, g/dl | 0.8812 (0.6454-1.2032) | 0.4262 |
| PRECISE DAPT-score | 1.0012 (0.9338-1.0734) | 0.9739 |

Abbreviations: aHR, adjusted hazard ratio; BARC, Bleeding Academic Research Consortium; CI; confidence interval; DAPT, dual antiplatelet therapy; h, hour; CABG, coronary artery bypass graft; BMI, body-mass index; GRACE, Global Registry of Acute Coronary Events; h; hours; PREdicting bleeding Complications In patients undergoing Stent implantation and subsequent Dual Antiplatelet Therapy.
